# Synthesis and Spatial Order Characterization of Controlled Silica Particle Sizes Organized as Photonic Crystals Arrays

**DOI:** 10.3390/ma15175864

**Published:** 2022-08-25

**Authors:** Silvia Adriana Estrada Alvarez, Isabella Guger, Jana Febbraro, Ayse Turak, Hong-Ru Lin, Yolanda Salinas, Oliver Brüggemann

**Affiliations:** 1 Institute of Polymer Chemistry, Johannes Kepler University Linz, Altenberger Strasse 69, 4040 Linz, Austria; 2 Linz Institute of Technology (LIT), Johannes Kepler University Linz, Altenberger Strasse 69, 4040 Linz, Austria; 3 Department of Engineering Physics, McMaster University, Hamilton, ON L8S 4L7, Canada; 4 Department of Chemical and Materials Engineering, Southern Taiwan University of Science and Technology, Nantai St. No.1, Tainan 71005, Taiwan

**Keywords:** silica particles, opals, polydispersity index, photonic crystals, disLocate, Voronoi tessellations, bond order parameters

## Abstract

The natural occurrence of precious opals, consisting of highly organized silica particles, has prompted interest in the synthesis and formation of these structures. Previous research has shown that a highly organized photonic crystal (PhC) array is only possible when it is based on a low polydispersity index (PDI) sample of particles. In this study, a solvent-only variation method is used to synthesize different sizes of silica particles (SiPs) by following the traditional sol-gel Stöber approach. The controlled rate of the addition of the reagents promoted the homogeneity of the nucleation and growth of the spherical silica particles, which in turn yielded a low PDI. The opalescent PhC were obtained via self-assembly of these particles using a solvent evaporation method. Analysis of the spatial statistics, using Voronoi tessellations, pair correlation functions, and bond order analysis showed that the successfully formed arrays showed a high degree of quasi-hexagonal (hexatic) organization, with both global and local order. Highly organized PhC show potential for developing future materials with tunable structural reflective properties, such as solar cells, sensing materials, and coatings, among others.

## 1. Introduction

The dimensional periodic arrangements capable of controlling photo propagation are known as photonic crystals (PhC) [1]. They have been found to be responsible for the coloration involving keratin formation for a variety of animals, such as in structures found in the butterfly wings [2] or guanin crystal arrangements in chameleon skin [3]. However, perhaps the best known natural occurring photonic crystals are precious opals, which consist of highly ordered silica particles [4]. Thus, there is significant interest in silica particles (SiPs) thanks to their natural occurrence and high biocompatibility. There are reports of different methods to fabricate SiPs, however among them, the typical approach is the Stöber method [5,6]. This well-known procedure utilizes the ammonia-catalyzed hydrolysis of silicon alkoxides, specifically tetraethyl orthosilicate (TEOS), in an alcohol solution, to produce the particles.

Since the first description of the reaction, there has been considerable effort made to uncover the factors affecting the final size of the particles and the conditions for high homogeneity [7]. The temperature [8,9] of the reaction as well as the concentration of the reagents have been investigated. However, in recent years it has been discussed that a key factor to the limits of the reaction lies in the nucleation process [10], which is highly sensitive to the order and time of the addition of TEOS, pH, vortex of reaction, and viscosity of solvent, among others.

Homogeneity of the final particles can be measured in terms of the polydispersity index (PDI), where less than 0.05 is considered monodisperse [11,12]. Low PDI is particularly important for the formation of highly organized arrays such as PhC, which have become of increasing interest due to their infinite applications where tunable structural reflective properties are required, such as biological sensors, medicine [13,14,15], structural colored materials, textiles [16,17,18], electronics, and solar cells [19,20].

Research on synthetic PhCs was advanced with the work of Yablonovich and John, who engineered and detailed three types of PhCs: one, two and three dimensional [21]. After this categorization, many different approaches to produce synthetic photonic crystal were developed [22]. The primary method used is the self-assembly of monodisperse particles, such as by sedimentation, vertical deposition, physical confinement, spin coating, and most recently the dip drawing method [23]. The aim of all these methods is to yield the most hexatic lattice as possible, which in a tridimensional level translates to the face-centered cubic (FCC) or the hexagonal close packing (HCP). Both configurations have the highest packing factor of 0.74. However, the FCC is the most round-like, which improves the prospect of the photonic band gap (PBG) [24].

The aim of the present study is to use an efficient and reproducible method of silica particle synthesis, that yields low PDI, such that the particles are suitable for the formation of highly organized photonic crystals arrays. Once the PhC are obtained, we propose a quantifiable way of characterizing their degree of organization via Voronoi tessellations and spatial statistical analysis, and provide further information about the hexatic lattice.

## 2. Materials and Methods

### 2.1. Chemicals

Tetraethyl orthosilicate (TEOS, 98%), and ammonia (NH3, 28% *w*/*w*) from Alfa Aesar (Karlsruhe, Germany). Ethanol (EtOH, ABS) used for the different stages of the synthesis and work-up was purchased from ChemLab (Zedelgem, Belgium). The water (18 MΩ cm pure water) was of Milli-Q Gradient system (Millipore Corp) to keep conditions constant. For the glass slides treatment, a 1:4 piranha solution (H_2_O_2_/H_2_SO_4_) was used. Hydrogen peroxide (33.3%) was purchased from VWR Chemicals and sulphuric acid (95–97%) was received from Merck. All reagents were used as received.

### 2.2. Instruments for Syntehsis and Sample Preparation

During the fabrication of the silica particles (SiPs), a syringe pump (KDS 100 Legacy Syringe Pump, KD Scientific Inc., sourced from Merck KGaA, Darmstadt, Deutschland) was used for the controlled addition rate of TEOS. After 3 h of stirring, the solution was centrifuged (Thermo Heraeus Multifuge 1S Centrifuge, Thermo Fisher Scientific Inc., Linz, Austria) for 15 min at 5000 rpm, and the same settings were used for washing the particles. An ultrasonic cleaner (with digital timer—VWR International, Vienna, Austria) was used during the different steps of the synthesis and the characterization, to fully disperse the particles. Before analysing the samples under the scanning electron microscopy (SEM), the fabricated silica particles and photonic crystals were gold coated (Sputter Coater and Carbon Coater for TEM/SEM, S series, Instrument Futurism, Linz, Austria) with an average layer of 7 nm.

### 2.3. Characterization Methods

Dynamic light scattering (DLS) (Zetasizer Nano ZSP from Malvern Instruments, Worcestershire, UK) measurements were carried out to determine the average hydrodynamic diameter (D_h_) and the polydispersity index (PDI). In this study, the PDI refers to the uniformity of the particles (where a value close to 0.0 is a perfect homogeneity of the sample, while a 1.0 is a sample with a large variety of sizes). The samples were prepared in 0.1 M solution with Milli-Q water as dispersant in disposable cuvettes (DTS 0012), sonicated for 30 min. The DLS measurements via intensity were performed at 25 °C without filtration before the measurements. SEM images were obtained with a Jeol 6400 (Jeol, Peabody, MA, USA). The optical observation of the SiPs was carried out with an optical microscope at ×200 magnification (ZEISS axio imager A1m—Carl Zeiss AG, Vienna, Austria). The reflection spectra were measured using a spectrometer (LAMBDA 1050+ UV/VIS/NIR Spectrometer with 150 mm InGaAs Int. Sphere and UV WinLab software—PerkinElmer Inc., Linz, Austria). To characterize the organized silica arrays, the same coating conditions and SEM were used. The degree of organization was determined with Voronoi tessellation, using Mathematica package **disLocate**, a suite of tools to rapidly quantify the spatial structure of a two-dimensional dispersion of objects [25].

### 2.4. Synthesis of SiPs

The SiPs were synthesized using the Stöber method [5], through the controlled hydrolysis and condensation of tetraethyl orthosilicate in an alcohol medium. With the purpose of maintaining all conditions uniform for all experiments, the only variant to the synthesis of the different sizes of SiPs was the amount of solvent used, i.e., ethanol, while the molar amount of ammonia and of water was kept constant in relation to the used TEOS. The amount of EtOH used were 100 mL, 80 mL, 60 mL, and 40 mL.

The different amounts of ethanol were mixed with 8 mL of ammonia (80 mmol) and 3 mL of water in a 250 mL round bottom flask at 60 °C for 10 min. 6 mL of TEOS (26 mmol) were added slowly with the addition pump set to 18 mL/h. Constant and controlled addition speed allows reproducibility of the particles size. After the full addition of all reagents, the reaction was stirred for 3 h and then stopped by centrifugation to prevent further nucleation. It was then washed three times with 50 mL of EtOH to remove all unreacted monomers. This synthesis yielded the-so-called samples SiPs100, SiPs80, SiPs60, and SiPs40, in relation to the amount of solvent used during their preparation.

### 2.5. Preparation of SiPs Based PhC

The opalescent PhC were prepared by means of the evaporative deposition self-assembly method using the suspension of the SiPs in EtOH. To start with, the glass slides were made hydrophilic by immersing them into a 1:4 piranha solution (H_2_O_2_/H_2_SO_4_) while stirring the solution for 1 h. The glass slides were then rinsed with several aliquots of Milli-Q water to remove the piranha solution. The slides were then dried with a stream of nitrogen. This treatment further activates the -OH groups on the surface of the glass.

**Figure 1:**
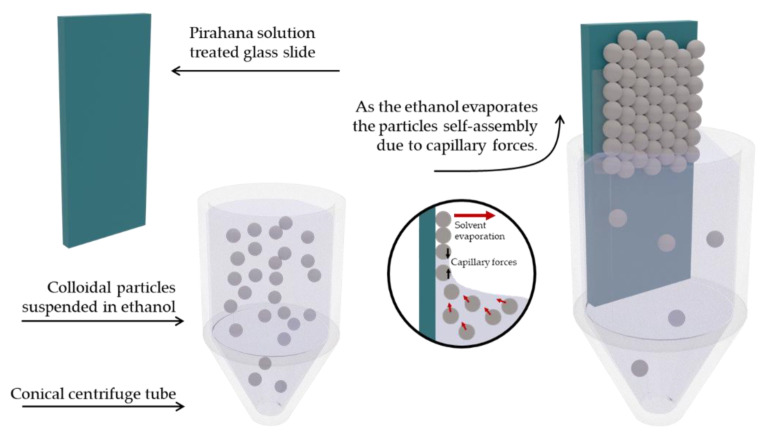
Setting to obtain organized an opalescent array of SiPs. The particles were fully suspended in ethanol at different concentrations, within a conical bottom centrifuge tube. The conical bottom was necessary to avoid a drastic thickening of the arrays at the bottom of the slide. A piranha solution treated glass slide is carefully placed vertically in the tube. The system is placed in a quiet, vibration free environment, where the ethanol evaporates at constant rate. The suspended particles self-assemble on the glass due to capillary forces in an organized manner as the evaporation takes places.

The glass slides were then inserted vertically in conical-bottom centrifuge tubes. Figure 1 exemplifies the formation of the opalescent films on the piranha solution treated glass. These tubes contain colloidal SiPs suspensions of the different concentrations (0.5%, 1%, 2% and 4% *w*/*w* of the SiPs). The conical shape of the tubes was necessary to prevent a thicker formation of opal on the bottom of the slide. This is expected to occur since the concentration of SiPs increases as the solvent evaporates. After the ethanol was evaporated completely, the SiPs organized array was obtained on both sides of the glass slides, which appeared as an opalescent structure.

## 3. Results and Discussions

To begin with, we synthesized a library of different sizes of silica particles. To keep control of the reproducibility, only the solvent amount was changed to obtain different silica particle sizes [26]. The SiPs were characterized via SEM and DLS, where the diameter and the hydrodynamic diameter (D_h_) were obtained; the latter here is usually a larger number, since it refers to moving particles in a solution. To ensure the quality of the subsequent PhC, only samples with PDI lower than 0.05 were considered. For the formation of the opalescent organized arrays, the particles of different sizes were dispersed in ethanol, which was later evaporated leaving the arrays of sedimented particles. This material was characterized by scanning electron microscopy (SEM). Additional to this characterization method, we analyzed the lattice using Voronoi diagrams, with the purpose of obtaining a quantifiable method of degree of organization.

### 3.1. Synthesis and Characterization of SiPs

By maintaining all conditions uniformly in all experiments and only varying the amount of solvent used, the reproducibility capability was maximized. This includes the use of the same oval stirrer as the different vortexes affected the nucleation and thus the final average particle size. The average SiPs diameter size was measured via SEM, and the average hydrodynamic diameter was measured via DLS. For significant validation of the results obtained from the SEM, 50 different points were measured of 5 different samples of SiPs for each experiment. In the case of DLS, 5 aliquots were measured from 15 different samples.

**Figure A1:**
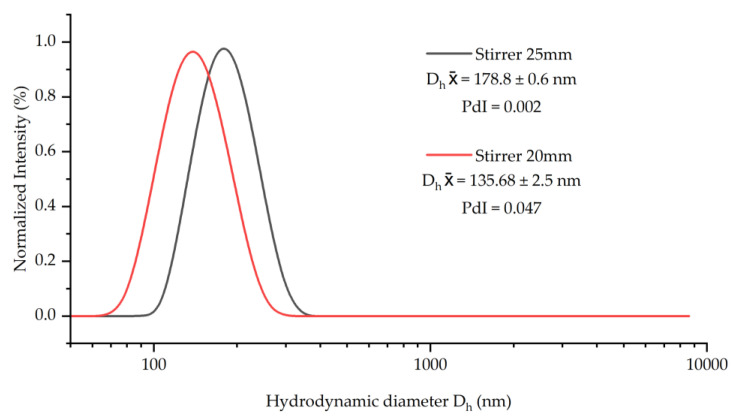
Hydrodynamic average diameter of particles measured via dynamic light scattering (DLS) using two different stirrer sizes. The different samples were fabricated with two sizes of stirrers, 20 and 25 mm. The vortex created by the stirrers resulted consistently in different particles sizes, although all the other conditions were carefully maintained constant.

As an example of the sensitivity of the reaction, Figure A1, of the appendix shows the hydrodynamic diameter obtained using 100 mL of solvent and a 20 mm oval stirrer as on average 135.6 ± 2.5 nm, however using a 25 mm oval stirrer the hydrodynamic diameter is 178.8 ± 0.6 nm. All the experiments carried out in this research were performed using a 25 mm oval stirrer.

The varying amounts of ethanol used were 100 mL, 80 mL, 60 mL, and 40 mL, which yielded an average particle diameter of 150 ± 3.1 nm, 233 ± 2.6 nm, 365 ± 2.4 nm, and 490 ± 4.3 nm, and a hydrodynamic average diameter (D_h_ $\bar{X}$) of 178.8 ± 0.6 nm, 242.7 ± 2.2 nm, 396.3 ± 2.3 nm, and 518.5 ± 4.2 nm for SiPs100, SiPs80, SiPs60, and SiPs40, respectively. Table 1 summarizes the experiments carried out, together with the results obtained from measuring the diameter (**⌀**) and hydrodynamic diameter (D_h_).

**Figure 2:**
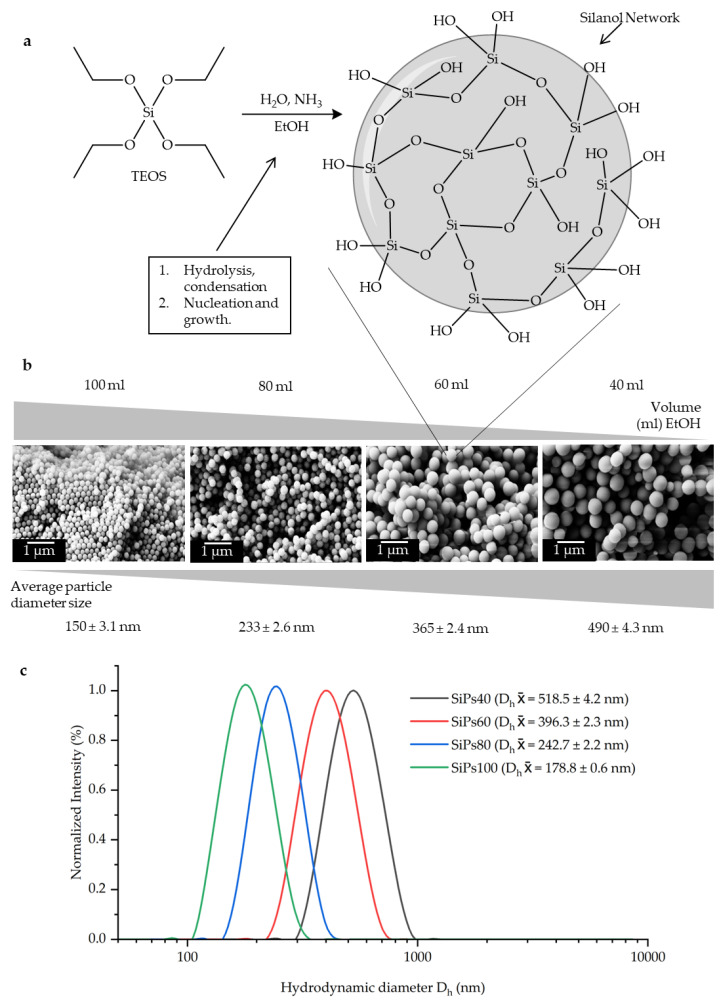
Synthesis and characterization of the SiPs. (**a**) Scheme of the SiPs synthesis. After the adhesion of TEOS to the ethanol solution, hydrolysis takes places promoting the formation of the silanol network. This in turn starts the nucleation for the formation of the SiPs. (**b**) SEM images of the obtained SiPs, and the representation of the inversely proportional relationship between the amount of EtOH and the final diameter of the SiPs. (**c**) Hydrodynamic sizes of the SiPs measured via DLS. The hydrodynamic diameter is usually a higher value than the diameter since it refers to moving particles in a solution.

The scheme in Figure 2a shows the reaction that takes place when synthesizing the SiPs from TEOS. After the start of the addition of TEOS to the reaction, hydrolysis takes place to form silanol monomers, which later condense giving way to a siloxane network, which allows the formation of nuclei and subsequently growth. This step takes place during the 3 h that the reaction was stirred. By consistently stopping the reaction at a set time, further formation of nuclei and growth is prevented, thus controlling the size of the synthesized SiPs while keeping a low PDI. Figure 2b represents the inversely proportional relationship that the amount of solvent has to the final size of particles and shows the respective SEM images.

A monodisperse sample of SiPs is necessary for a highly organized lattice; this required the PDI to be lower than 0.05. This index was measured together with the D_h_ using DLS. The results show that the previously explained procedure for the synthesis of the SiPs yields particles with the desired range of PDI. All samples used for the consecutive organizing sedimentation showed a PDI between 0.002 and 0.05. Figure 2c shows the graphs obtained from the DLS analysis displaying the D_h_ and the PDI.

### 3.2. Formation and Characterization of the SiPs PhC

The opalescent organized arrays were formed using different initial concentrations of SiPs in ethanol (0.5%, 1%, 2%, and 4% *w*/*w*). The solvent evaporation method used for the formation of the opals on the glass slides (2 × 2 cm), translated to a constant increase of the suspended particles concentration as the solvent evaporated, because of -this, all the samples were thicker on the lower part than on the upper part. Therefore, the thickness considered for this study was only taken from the middle of the sample.

The following two subsections explore the characterization of the PhC via SEM and the degree of organization with the help of statistical spatial order parameters.

#### 3.2.1. Visual Characterization

**Figure 3:**
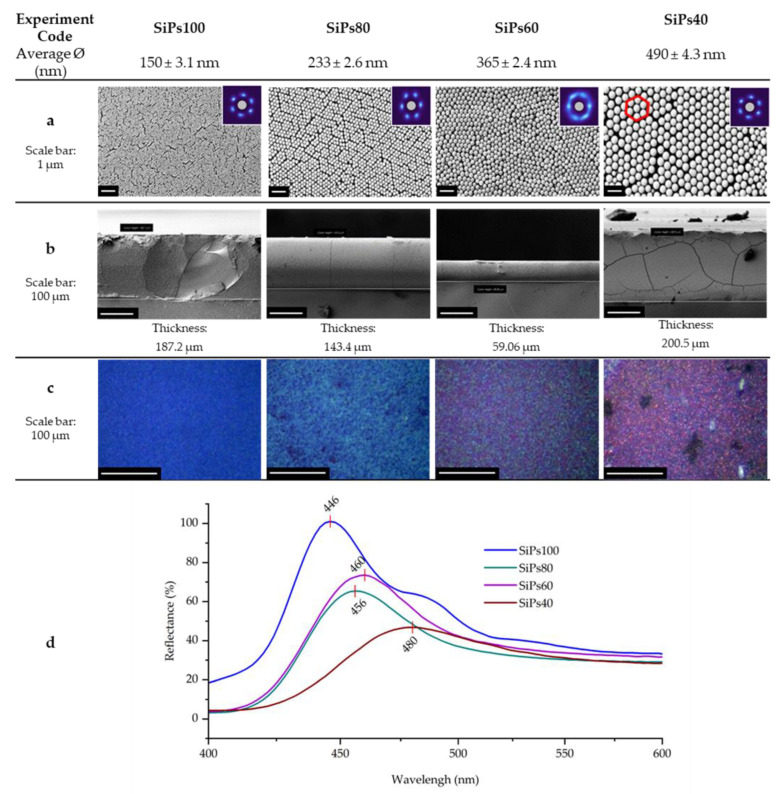
SEM and optical microscope observation of the SiP photonic crystals and their reflected wavelengths. The samples used for these figures were made using 1% of suspended particles in EtOH via solvent evaporation method. (**a**) SEM top view of the silica particle based photonic crystals, the inserts in the images show the correlation map or fast Fourier transform. These self-assembled organized layers were formed after the evaporation of the solvent. Qualitatively, they show a high level of hexagonal organization, as marked in the red frame. (**b**) SEM side view of the formed PhC. The thickness of the layers does not seem to follow any trend related to the particle size. (**c**) Optical microscope view of the same samples showing different colors. (**d**) The reflected wavelength that aligns with the results of part c. The wavelength of SiPs80 and SiPs60 are very close, which explains the similar coloration seen in the optical microscope.

SEM was used for characterizing the top and side view of the formed PhC. The top view shows the morphology and the high degree of organization. Visually it is possible to recognize the hexagonal pattern formed by the SiPs (shown in Figure 3 on the SEM top view of the SiPs40). This is a characteristic trait of the FCC and HCP arrangement. This is also supported by the coordination cloud or fast Fourier transform (FFT) of the image, pictured in the insert in the top right-hand corner (Figure 3a), showing six distinct equally spaced lobes as expected for good hexagonal close packing. SiPs60 have less distinct lobes, suggesting that it is less well ordered than the other three films. 

These kinds of organization are the most desirable for photonic crystals since a close packing factor is most suited for wavelength propagation. A further examination and quantifiable reasoning of the hexagonal configuration of the SiPs is found on the next section.

The thickness of the formed opals was not found to follow a specific pattern and did not seem to be influenced by the particle size. Our reasoning to this occurrence is that after a certain number of stacked layers, the integrity is compromised due to weight causing the most recently sedimented layers to slide down possibly bringing some of the older sedimented layers with them. This occurrence also explains the source of cracks seen specially on the bigger particles (Figure 3b, SEM side view of the SiPs40).

By placing the samples in an optical microscope, it was possible to observe the reflected wavelength, of which the source is the microscope beam. The smallest particles (SiPs100) reflected an intense homogeneous blue, similar to what is commonly known as “cobalt blue”. The second smallest particles showed shades of green combined with domains of blue, giving up a color that could be describe as “dark teal”. The SiPs60 give up a more purple like appearance as it shows domains of blue, and at the same time of red, this could be recognized as a “cyber grape” appearance. The biggest particles reflected a darker red like tone that could be seen as a “plum” color. Similar visual coloration was obtained by W. Gao [26], however their method of self-assembly was gravitational sedimentation, instead of evaporation deposition.

This color observation matched the results obtained from the UV/Vis spectrometer, shown Figure 3d. For this characterization, the samples were place horizontally in the machine for the measurements. The closeness of the measured wavelengths of SiPs60 and SiPs80 is attributed to the blue domains found in both samples.

#### 3.2.2. Spatial Statistics Characterization

To describe quantitatively the structure and assign a value to the relative order, the formed PhC arrays were characterized from SEM micrographs with spatial statistics obtained using the Mathematica package, **disLocate** (**D**etecting **I**ntermolecular **S**tructure **Locate**d at particle positions) [25].

Order on the local scale can be sub-categorized into three distinct types: translational, entropic and angular. Translational order occurs when every particle in the system has an exact position that repeats at a specific distance, defined by a specific translation period.

Entropic order is achieved when the amount of free volume (sections unoccupied by particle mass) encompassed by the system is the lowest possible such that the system is at maximum density. Angular order is related to the relative arc-separation of the “bonds” or touching contacts between a particle and its neighbors.

Complete periodicity is seen when the neighbors for any particular particle have the same angular arc symmetry, as well as being equidistant, with maximized covering area due to the equivalent position of each particle relative to all others. If any one of these types of order are not met, the system can be considered to be in a mesophase [27], such as that observed for plastic crystals (limited orientational or rotational order, but long-range translational order [28]) or liquid crystals (limited translational order but long-range angular order) [29].

**Figure 4:**
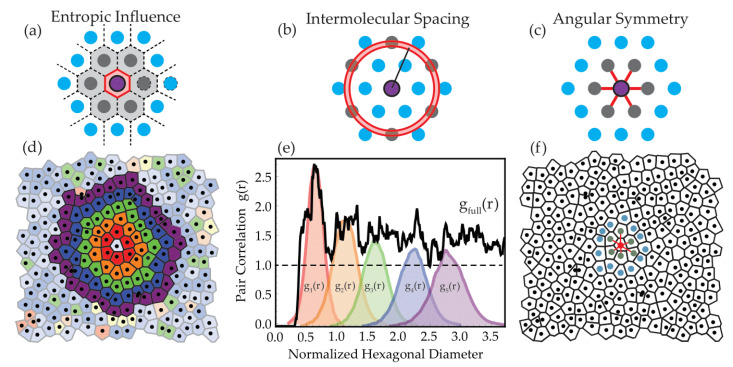
Schematic of order metrics used to characterize the morphology located at particle positions: (**a**) Voronoi tessellations (**b**) pair correlation function (**c**) bond order parameter, for each category of order possible in a 2D pattern. (**d**) Voronoi tessellation of a planar pattern of particles with coloring scheme representing the Voronoi shell description of neighbors. The coloring scheme of outlier cells reflects the deviation of covering area from that of a hexagonal lattice. (**e**) The pair correlation function separated by the Voronoi shells, showing the connection between local and global order metrics. The sum of finite shellular descriptions leads to an approximation of the configurational average g(r). At large values, the value of the pair correlation goes to uniform probability (a value of 1 shown by the dotted horizontal line). (**f**) Voronoi tessellation of a planar pattern of particles showing the “bonds” connecting adjacent particles, where the angle between bonds compared against the expected angle for hexagonal packing can be used to determine the local bond order for each cell, as well as the ensemble average global bond order.

To assess each type of order for the silica particles opal arrays, different spatial order metrics were applied to the images, as shown schematically in Figure 4.

Local free volume and the complementary metric, covering area [30] can be calculated by partitioning the substrate into a Voronoi tessellation around each individual particle (Figure 4a,d) [31]. To define each cell of the tessellation, a perpendicular line at the midpoint along the line-of-sight vector connecting nearest neighbors around every particle is calculated. The intersection of these lines defines the unit cell surrounding each particle. The covering area is then extracted and compared to that expected for a hexagonally close-packed system, to show the distribution of hexagonally packed regions. As there is an entropic driving force that aligns faceted or functionalized particles so as to maximize the system entropy by minimizing the free volume, analogous to chemical valence states [32,33,34], it is possible to define a coordination number from the number of Voronoi cell facets which contain the particle (see Figure 4d). To show the various local order states, the Voronoi tessellation maps can be colored by the cell area deviation from that expected for perfect hexagonal packing, by the coordination number, or by the deviation of the bond order parameter from the expected symmetry value (discussed below).

Going beyond the first neighbor, it is possible to quantify the positional order probabilistically using the pair correlation function [35]. The pair correlation function *g(r)* describes the number of objects within a small shell at a distance away from a central particle and averaged over all particles in the system (Figure 4b,e). Imagining the neighbor distances as a series of Voronoi cells radiating out from each particle, the ensemble sum of each Voronoi cell leads to an approximation of the configurational average radius *(r)*. In a periodic lattice, the objects are evenly spaced out at specific distances by lateral translations of a repeated unit (unit cell). The degree of order can be determined by the number of peaks in the pair correlation function, with predictable spacing between peaks for each Bravais lattice. At large distances, the pair correlation function converges to uniform probability (shown by the horizontal dotted line in Figure 4e). The convergence of the pair correlation function is related to the extent of spatial periodicity, with high periodicity showing many multiple shells before there is equal chance of finding a particle at any distance. By contrast, a system with complete spatial randomness would have a uniform probability for all values of distance, above the nearest neighbor distance (or particle diameter for particles in contact).

**Figure A2:**
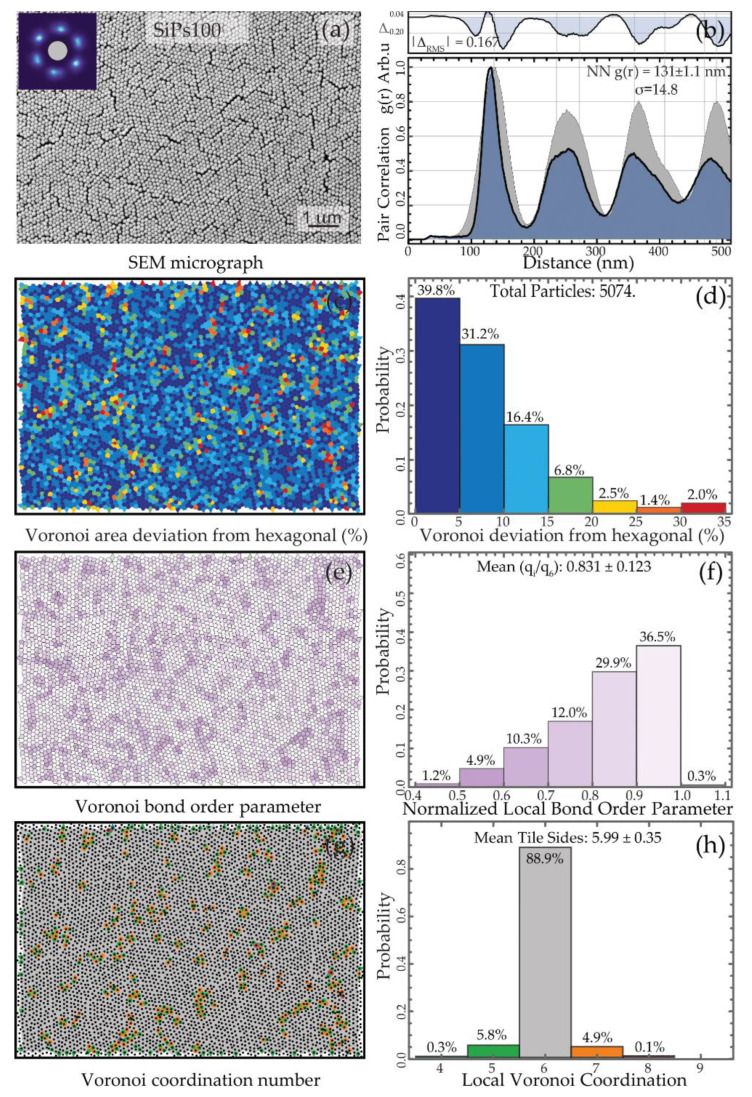
Spatial statistics of sedimented SiPs100. (**a**) SEM image with insert of coordination map or fast Fourier transform (FFT) showing six distinct lobes as expected for hexagonally packed systems. (**b**) pair correlation function for the particle distributions (blue) and that of the matched hexatic lattice (grey). Using the average spacing (2r_hex_) of a hexagonal lattice with the same number density (located at particle positions), the matched hexatic lattice is determined by randomly displacing the particles from their center with a mean distance relative to a normal probability distribution. The widths of these peaks correspond to the average mean displacement each particle has relative to this expected spacing, described by the lattice disorder parameter, σ. The nearest neighbor distance can be determined from the maximum of the first peak in the *g(r)*. The insert above shows the difference spectrum, which is the subtraction between the pair correlation function and the hexatic lattice. (**c**) Voronoi tessellations of the particle centroids colored by percent deviation of the expected cell area produced from hexagonal packing. (**d**) Histogram of the probabilities of various percent deviations of the Voronoi cell areas from those produced from hexagonal packing. Taking into account cells with packing of less than 15% of a perfect hexagonal lattice (three blue bars), the film shows ~87% hexagonal packing regions. (**e**) Voronoi tessellations of the particle centroids colored by the normalized bond order parameter. Whiter areas indicate particles that have high angular order in a hexagonal (*q_6_*) symmetry basis. (**f**) Histogram of the probabilities for various values of the bond order parameter. The extracted global bond order parameter is 0.831 *q/q6* (i.e., 83% of perfect hexagonal symmetry). Expectation value for individual cells (maximum of histogram) is 90% of hexagonal symmetry (**g**) Voronoi tessellation of the particle centroids colored by the number of shared facets (coordination number). (**h**) Histogram of the probabilities for each coordination number. Almost 89% of particles have six nearest neighbors, with only ~5% of matched 5/7 declination pairs.

**Figure A3:**
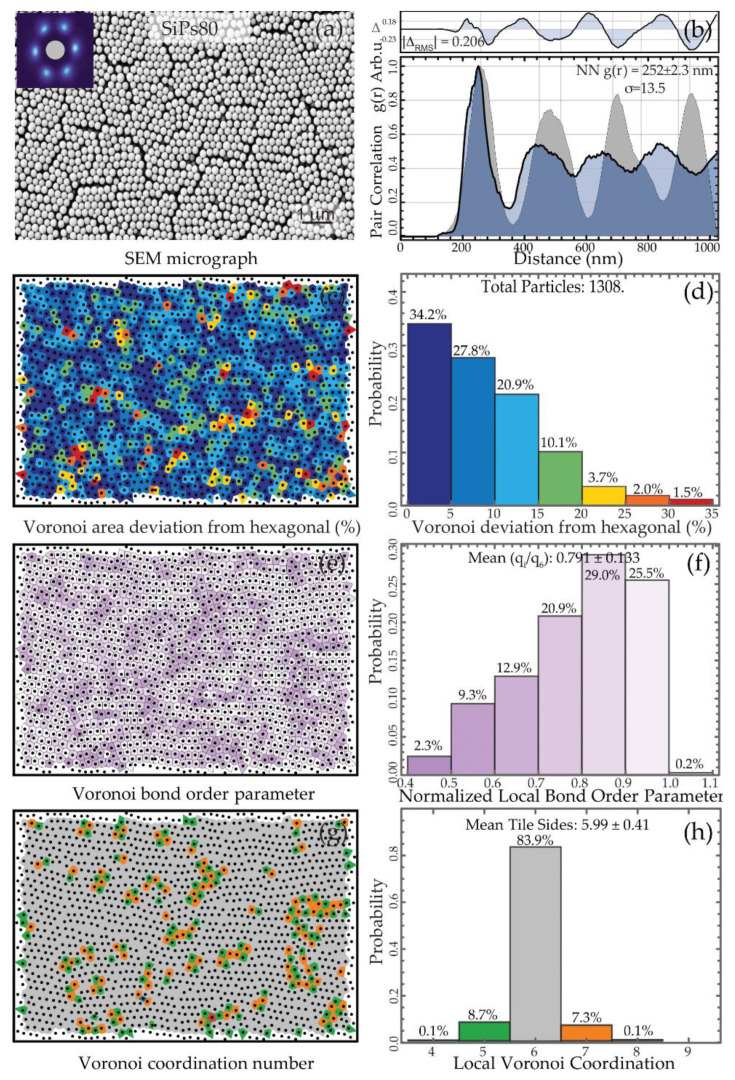
Spatial statistics of sedimented SiPs80. (**a**) SEM image with insert of coordination map or fast Fourier transform (FFT) showing six distinct lobes as expected for hexagonally packed systems. (**b**) pair correlation function for the particle distributions (blue) and that of the matched hexatic lattice (grey). Using the average spacing (2r_hex_) of a hexagonal lattice with the same number density (located at particle positions), the matched hexatic lattice is determined by randomly displacing the particles from their center with a mean distance relative to a normal probability distribution. The widths of these peaks correspond to the average mean displacement each particle has relative to this expected spacing, described by the lattice disorder parameter, σ. The nearest neighbor distance can be determined from the maximum of the first peak in the *g(r)*. The insert above shows the difference spectrum, which is the subtraction between the pair correlation function and the hexatic lattice. (**c**) Voronoi tessellations of the particle centroids colored by percent deviation of the expected cell area produced from hexagonal packing. (**d**) Histogram of the probabilities of various percent deviations of the Voronoi cell areas from those produced from hexagonal packing. Taking into account cells with packing of less than 15% of a perfect hexagonal lattice (three blue bars), the film shows ~83% hexagonal packing regions. (**e**) Voronoi tessellations of the particle centroids colored by the normalized bond order parameter. Whiter areas indicate particles that have high angular order in a hexagonal (*q_6_*) symmetry basis. (**f**) Histogram of the probabilities for various values of the bond order parameter. The extracted global bond order parameter is 0.791 *q/q6* (i.e., 79% of perfect hexagonal symmetry). Expectation value for individual cells (maximum of histogram) is 80–90% of hexagonal symmetry (**g**) Voronoi tessellation of the particle centroids colored by the number of shared facets (coordination number). (**h**) Histogram of the probabilities for each coordination number. Almost 84% of particles have six nearest neighbors, with ~8% of matched 5/7 declination pairs.

**Figure A4:**
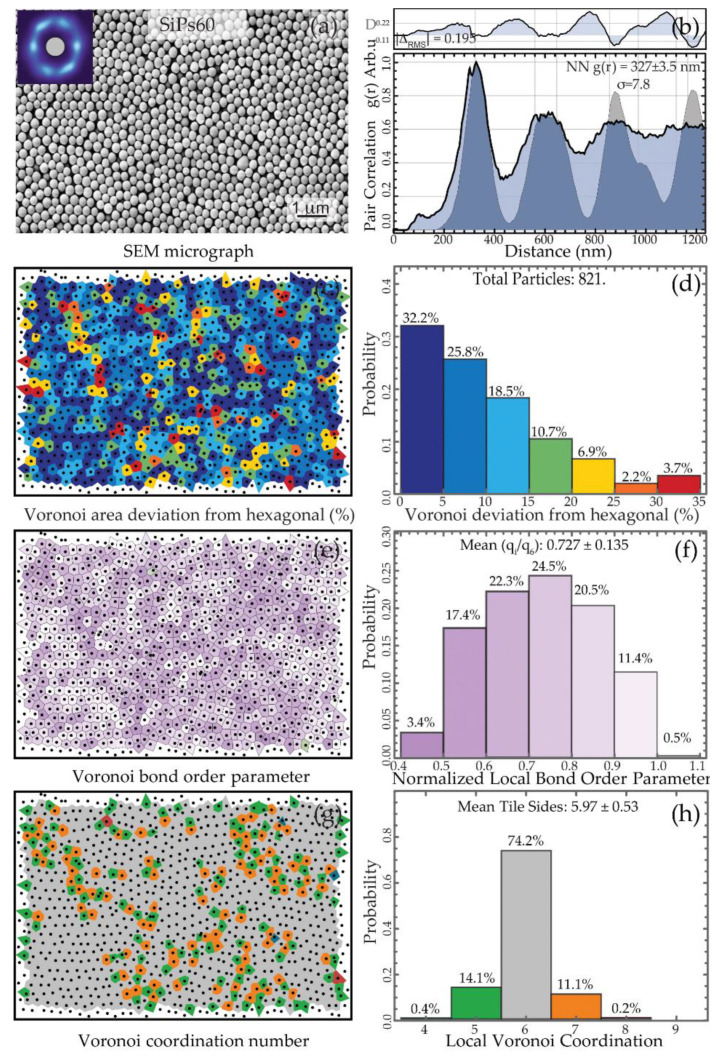
Spatial statistics of sedimented SiPs60. (**a**) SEM image with insert of coordination map or fast Fourier transform (FFT) showing six lobes as expected for hexagonally packed systems. However, the smearing suggests a more powder like disordered configuration rather than sharp lobes. (**b**) pair correlation function for the particle distributions (blue) and that of the matched hexatic lattice (grey). Using the average spacing (2r_hex_) of a hexagonal lattice with the same number density (located at particle positions), the matched hexatic lattice is determined by randomly displacing the particles from their center with a mean distance relative to a normal probability distribution. The widths of these peaks correspond to the average mean displacement each particle has relative to this expected spacing, described by the lattice disorder parameter, σ. The nearest neighbor distance can be determined from the maximum of the first peak in the *g(r)*. The insert above shows the difference spectrum, which is the subtraction between the pair correlation function and the hexatic lattice. (**c**) Voronoi tessellations of the particle centroids colored by percent deviation of the expected cell area produced from hexagonal packing. (**d**) Histogram of the probabilities of various percent deviations of the Voronoi cell areas from those produced from hexagonal packing. Taking into account cells with packing of less than 15% of a perfect hexagonal lattice (three blue bars), the film shows 76.5% hexagonal packing regions. (**e**) Voronoi tessellations of the particle centroids colored by the normalized bond order parameter. Whiter areas indicate particles that have high angular order in a hexagonal (*q_6_*) symmetry basis. (**f**) Histogram of the probabilities for various values of the bond order parameter. The extracted global bond order parameter is 0.727 *q/q6* (i.e., 73% of perfect hexagonal symmetry). Expectation value for individual cells (maximum of histogram) is 70–80% of hexagonal symmetry (**g**) Voronoi tessellation of the particle centroids colored by the number of shared facets (coordination number). (**h**) Histogram of the probabilities for each coordination number. Almost 74% of particles have six nearest neighbors, with ~13% of matched 5/7 declination pairs.

**Figure A5:**
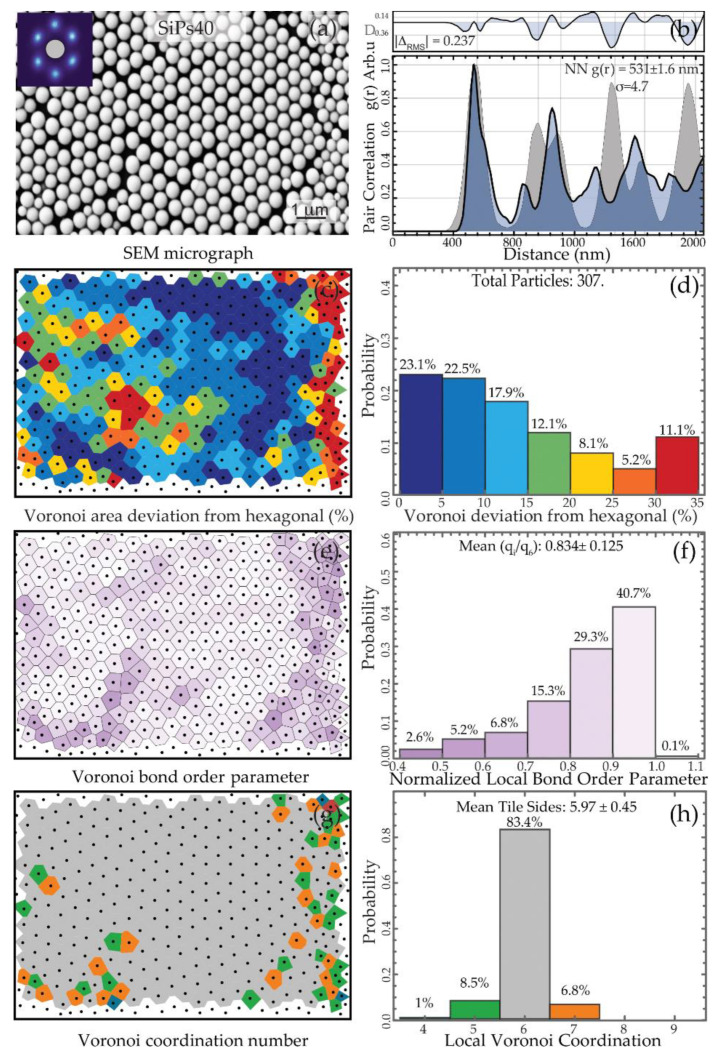
Spatial statistics of sedimented SiPs40. (**a**) SEM image with insert of coordination map or fast Fourier transform (FFT) showing six distinct lobes as expected for hexagonally packed systems (**b**) pair correlation function for the particle distributions (blue) and that of the matched hexatic lattice (grey). Using the average spacing (2r_hex_) of a hexagonal lattice with the same number density (located at particle positions), the matched hexatic lattice is determined by randomly displacing the particles from their center with a mean distance relative to a normal probability distribution. The widths of these peaks correspond to the average mean displacement each particle has relative to this expected spacing, described by the lattice disorder parameter, σ. The nearest neighbor distance can be determined from the maximum of the first peak in the *g(r)*. The insert above shows the difference spectrum, which is the subtraction between the pair correlation function and the hexatic lattice. (**c**) Voronoi tessellations of the particle centroids colored by percent deviation of the expected cell area produced from hexagonal packing. (**d**) Histogram of the probabilities of various percent deviations of the Voronoi cell areas from those produced from hexagonal packing. Taking into account cells with packing of less than 15% of a perfect hexagonal lattice (three blue bars), the film shows 63.5% hexagonal packing regions. This is likely due to the disorganized region visible at the right hand side. (**e**) Voronoi tessellations of the particle centroids colored by the normalized bond order parameter. Whiter areas indicate particles that have high angular order in a hexagonal (*q_6_*) symmetry basis. (**f**) Histogram of the probabilities for various values of the bond order parameter. The extracted global bond order parameter is 0.834 *q/q6* (i.e., 83% of perfect hexagonal symmetry). Expectation value for individual cells (maximum of histogram) is 90% of hexagonal symmetry (**g**) Voronoi tessellation of the particle centroids colored by the number of shared facets (coordination number). (**h**) Histogram of the probabilities for each coordination number. Over 84% of particles have six nearest neighbors, with ~8% of matched 5/7 declination pairs.

Angular orientation order describes the likelihood of finding an object at a given angular arc-separation between neighboring particles, most commonly thought of as the symmetry state of a system. Angular order can be calculated by using the bond order parameter [36], which compares the angle between the central particle and its closest neighbors against a specified set of symmetry basis vectors (Figure 4c,f). In the aggregate across all particles, this can be collapsed down into a coordination cloud or fast Fourier transform (FFT) of the image [37,38]. For close-packed systems of circles, hexagonal configurations have the highest density, so the 6-fold bond order values (*q_6_*) are the most crucial ones. All of these methods have been widely used to characterize disorder, identify polycrystalline and disordered sections, and extract the probability of intermolecular spacings [39,40]. The complete spatial statistic description for each of the formed PhC arrays are summarized in Figure A2, Figure A3, Figure A4 and Figure A5.

**Figure 5:**
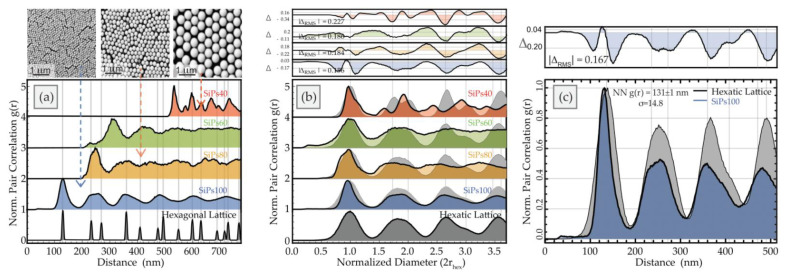
Pair correlation functions *g(r)* of centroids obtained from SEM images of PS inverse opals. Colored arrows show the corresponding SEM image resulting in the pair correlation function (**a**) Measurements of objects shows that the peak positions are misaligned. This confirms how the average spacing between particles is inversely correlated with the amount of solvent addition. The expected *g(r)* for a hexagonal lattice with similar intensity (average particles per unit area) as SiPs100 is shown for comparison. (**b**) Normalizing the distance to the average spacing (2r_hex_) of a hexagonal lattice with the same number density (located at particle positions) collapses the distributions into a shared spatial reference frame where peak positions can easily be compared. The smallest and largest diameter particles show correlation with the hexagonal lattice of similar intensity (average particles per unit area) for more than 4 degrees of order (peaks in the correlation function). For the intermediate diameters only one or two degrees of order are observed, indicating a larger amount of disorder in those systems. (**c**) The differences in *g(r)* are shown as the grey sections on an overlay of pcf from SiPs100 particle distributions and that of the matched hexatic lattice. The widths of these peaks correspond to the average mean displacement each particle has relative to this expected spacing. The inserts above (**b**,**c**) show the difference spectrum, which is the subtraction between a pair correlation function and the hexatic lattice.

Figure 5 shows the pair correlation function for the particle centroids for the SiPs arrays. As can be seen in Figure 5a, the pair correlation functions are misaligned for the various arrays, confirming that the average spacing between particles is inversely correlated with the amount of solvent addition. Normalizing the distance to the average spacing (2r_hex_) of a hexagonal lattice with the same number density of particles (located at particle positions), as seen in Figure 5b, collapses the distributions into a shared spatial reference frame where peak positions can easily be compared.

For SiPs80 and SiPs40, the nearest neighbor distance (maximum of the first peak of the pair correlation) is slightly larger than the average particle diameter (252 ± 2.3 nm and 531 ± 1.6 nm, respectively), consistent with the visible cracks observed in various regions increasing the average distance between particles.

For SiPs60, where there are no visible cracks, this spacing is smaller than the particle diameter (327 ± 3.5 nm), and there is even a small feature before the first peak around 100 nm. Typically for touching close-packed particles in a single layer, it is not possible for the particles to be closer than the particle diameter, and the pair correlation function is zero for values of distance less than the mean particle size. These features for SiPs60 suggest that there is some vertical disorder in the film, and what is visible in the SEM image is more than the top layer of the film. This is supported by the significantly thinner film thickness observed for this solvent addition. With densification of the film, particles have greater disorder vertically, and appear to pack more tightly. Overall, this film appears to be more disordered, with the pair correlation function having only two broad peaks before becoming featureless and converging on uniform probability. This type of behavior is typically associated with mostly disordered films, with a small degree of short-range order. This is also consistent with the coordination cloud or fast Fourier transform (FFT) shown in Figure 3, where the six lobes showing hexagonal packing are smeared, suggesting a loss of angular order. The higher level of disorder in this film could explain the inhomogeneous coloration for SiPs60, with both red and blue patches and even darkened regions visible. The periodicity is disrupted, and the reflected color is non-uniform.

Though SiPs100 also have an average spacing (131 ± 1.1 nm) less than the predicted particle diameters, the features of the pair correlation function are very different. Unlike for SiPs60, this film shows good correlation with a hexagonal lattice of similar intensity (average particles per unit area) for more than 6 degrees of order (peaks in the correlation function). The average spacing is consistent with that of a hexagonal lattice, as shown in Figure 5a. However, there is some positional disorder from a perfect hexagonal close packing, shown by a broadening of the peak widths. Slight deviations of the particle positions close to the global lattice positions will not change the mean particle spacings, but the small fluctuations increase the chance for neighbors to be found at slightly different distances, yielding peak broadening. Using our tool set, we can determine the extent of these fluctuations using an “hexatic” lattice, determined through an ensemble average of a hexagonal lattice matched to the particle spacing which has been modified by randomly displacing the particles from their center with a mean distance relative to a normal probability distribution [23]. In this way, the lattice retains the angular symmetry of the hexagonal lattice but relaxes the condition of maximal density. An overlay of the pair correlation function from SiP100 particle distributions and the matched hexatic lattice is shown in Figure 5c. The widths of the peaks correspond to the average mean displacement each particle has relative to its expected spacing. The insert above shows the difference spectrum, which is the subtraction between a pair correlation function and the hexatic lattice. These comparisons are also shown in Figure 5b. For films other than SiPs60, the pair correlation function shows good correlation with a hexatic lattice, with SiPs40 also showing a high degree of order with many peaks in the correlation function.

**Figure 6:**
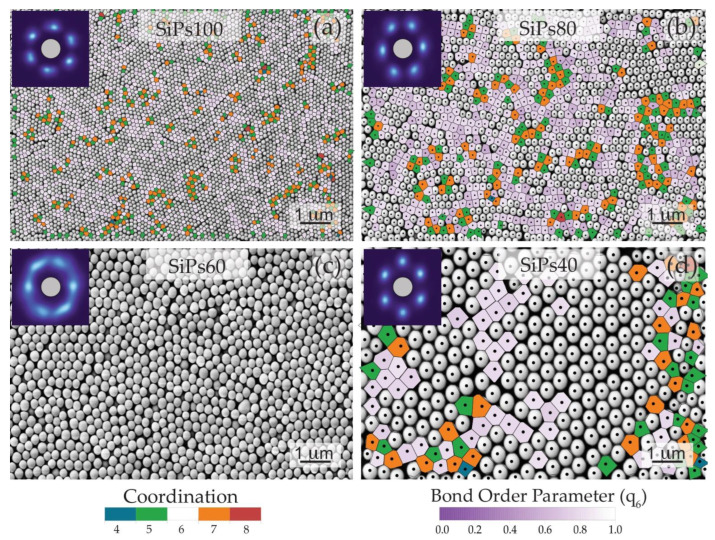
Overlay of Voronoi tessellation of the particle centroids over the corresponding SEM image of the particles for (**a**) SiPs100 (**b**) SiPs80 (**c**) SiPs60 and (**d**) SiPs40. Cells are colored by the number of shared facets and by the normalized bond order parameter for the type of symmetry for six neighbored cells. Whiter areas indicate particles that have high angular order in that symmetry basis. Particles with 5 and 7 neighbors typically highlight the grain boundaries and dislocation lines in their respective symmetry basis, but are not consistent with the cracks visible in the SEM images. Incorporating normalized bond order between 60% and 90% of that expected for a hexagonal lattice for 6 neighbor domains highlights the cracking observed in the films. SiPs60 in part c showed no correlations.

A critical feature of the hexatic lattice is that each particle has six-fold symmetry, with six nearest neighbors. This local order can be observed with Voronoi tessellations of the films (see Appendix A Figure A2, Figure A3, Figure A4 and Figure A5). All four systems show greater than 70% regions with Voronoi cells of high hexagonal entropic order (uncolored cells in Figure 6), within a larger area that is not globally perfectly hexagonal, (as already seen from the pair correlation analysis). This suggests roughly hexagonal symmetry arrangements, but with some relaxation of strict hexagonal packing. With defects or local disorder, the steric frustration at the boundaries between large hexagonal sections typically forms pairs of Voronoi cells with alternating 5 and 7 sides (green and orange regions). These are the so-called “disclination” defects [7], where the local number of neighbors is violated. In an analogy with dislocations, which are positional defects, a collection of 5/7 declination pairs are used to define “grain boundaries” between entropically ordered sections of 2D colloidal crystals [41,42]. The visible spacing or cracks between particles in certain regions for the films do not correspond well to the 5/7 declination pairs. This suggests that the cracks are not low-angle grain boundaries. They disrupt the periodicity of the lateral spacing (i.e., are not close-packed) but retain high hexagonal order, as would be expected for a hexatic system.

The bond order showcases the regions that have the correct angular spacing but lack translational symmetry, i.e., that are hexatic rather than perfectly hexagonal. The global *q_6_* bond order yields the average hexagonal nature of the film as a whole, and the local bond order shows how close each Voronoi cell is to being perfectly hexagonal. For the SiPs films, the global bond order parameter scales as SiPs60 < SiPs80 < SiPs100 < SiPs40. The values for SiPs100 and SiPs40 are similar, likely to within error. This behavior is supported by the pair correlations shown in Figure 5b, where both SiPs100 and SiPs40 showed regular peaks, with good correlation to a matched hexatic lattice, though SiPs40 seemed less so. This can be explained through the Voronoi analysis, where there is a region on the right-hand side of the image which contains a higher number of defects than the rest of the film. As the particle density is much lower for the SiPs40 film compared to SiPs100, the mean global values are more affected by such outliers with a smaller overall number of particles. For systems of similar intensity, the global values would be expected to be similar for both films. The lower value of global bond order for SiPs80 is likely due to the presence of more cracks in the system (as discussed below). The outlier is the SiPs60 film, which is the least hexatic (only 73% as much as a pure hexagonal system).

The localized bond order of each particle is lower in the crack regions, separating highly ordered domains where the local bond order is higher (white domains in Figure A2, Figure A3, Figure A4 and Figure A5e,f). For SiPs100 and SiPs40, the local bond order between 0.8 and 0.9 roughly follows the regions where cracks are visible in the film, when combined with the 5/7 neighbor domains, as shown in Figure 6. These sections separate the larger domains where there is almost perfect hexagonal packing. SiPs40 appears slightly more ordered than SiPs100 (crack domains 33% vs. 39%, respectively), but this value could be skewed by the low number of particles. Likely within error, both structures are similarly ordered.

The SiPs80 film requires a combination of 5/7 domains plus 6-fold domains with local bond order down to 60% of that expected for a pure hexagonal lattice to accommodate (imperfectly) the cracks. These together with the 5/7 declination pairs make up 56% of all domains. There seem to be more cracks visible in this film compared to SiPs100 and SiP40s, and those crack regions are more disordered.

The SiPs60 film has no correlation, as the overall film is disordered, rather than ordered with cracks. This is also supported by the FFT (coordination cloud) which is smeared out for SiPs60, compared to the bright six-fold distinct lobes for the other solvent additions.

Overall, the spatial statistical analysis of the different opalescent films suggests that SiPs100 and SiPs40, with the smallest and largest diameter particles, are similarly ordered. They consist of large hexatic domains, separated by cracks in the film that are not acting as grain boundaries, but show some loss of angular symmetry. The SiPs40 film is slightly less close-packed, with the average spacing between particles larger than the measured particle diameters. SiPs80 is less ordered, with more visible cracks and a greater number of Voronoi domains that have both relaxed translational and angular periodicity. However, the film is uniform with hexatic ordered domains between the cracked regions. SiPs60 has some short-range order but lacks both translational and angular periodicity over much of the film. Unlike the other SiPs films, it is likely also disordered vertically, without clear layered lattice-like planes, as the SEM image from the top of the film likely shows more than one layer, resulting in a thinner film and inhomogeneous opalescence.

## 4. Conclusions

The different sizes of silica particles were synthesized by only changing the amount of solvent used. This suggests that, although the reaction between ammonia and TEOS remains the same, the concentration of both reagents is changed. This, in turn, caused the growth process to be affected. In the case of 100 mL of EtOH used, there was a large nucleation, but the growth was diminished due to the distances between nuclei. In contrast, with a higher concentration, as in the case of the 40 mL of EtOH, the growth was enhanced due to the proximity of the forming silanol network. By closely monitoring the rate of addition of TEOS, we promoted a constant supply of the reagent and a controlled consumption of the same. This was the substantial reason for the low PDI, which is a key point for the formation of the PhC.

During the formation of the organized arrays on the piranha-solution-treated glass slides, it was of key importance that the evaporations took place in quiet, vibration free environment. These little disturbances cause wavelike shapes to appear on the dried PhC, which showed different thickness and proved unreliable for characterization. The assembly of the particles on the slides is largely due to the capillary forces at the meniscus area of the evaporating solvent and the glass slide.

The spatial statistics of the organized arrays yielded a quantifiable comparison of the degree of spatial, angular, and entropic organization. By simple visual observation, it is possible to perceive some hexagonal packing, typical of FCC and HCP lattices, however it is very difficult to determine to what extent and be able to compare different pathways of organizing particles, especially when the experiments are similar, and the positional differences are similar. In this study, we were able to observe that both 100 mL of EtOH and 40 mL of EtOH addition yielded highly organized films, with good correspondence to a quasi-hexagonal (hexatic) lattice, where some positional symmetry is relaxed, but with high angular symmetry, resulting in good opalescent films. The addition of 60 mL of EtOH resulted in a more disorganized film, with only 73% the character of a hexagonal close-packed lattice, with some loss of periodicity in the vertical direction as well, yielding non-uniform reflectance. The use of such statistics can drive the formation of highly ordered self-assembled arrays.

## Figures and Tables

**Table 1:** Impact of the different amounts of EtOH used for the synthesis of SiPs on the diameter.

Experiment Code	EtOH (mL)	Average Diameter ^1^ ⌀ (nm)	Hydrodynamic Average Diameter ^2^ D_h_ (nm)	PDI ^2^
SiPs100	100	150 ± 3.1	178.8 ± 0.6	0.002
SiPs80	80	233 ± 2.6	242.7 ± 2.2	0.020
SiPs60	60	365 ± 2.4	396.3 ± 2.3	0.022
SiPs40	40	490 ± 4.3	518.5 ± 4.2	0.034

^1^ Measured via SEM. ^2^ Measured via DLS.
